# DLX3 Inhibits the Proliferation of Human Dental Pulp Cells Through Inactivation of Canonical Wnt/β-Catenin Signaling Pathway

**DOI:** 10.3389/fphys.2018.01637

**Published:** 2018-11-20

**Authors:** Yunyan Zhan, Xiaoyan Li, Xiaohui Gou, Guohua Yuan, Mingwen Fan, Guobin Yang

**Affiliations:** ^1^The State Key Laboratory Breeding Base of Basic Science of Stomatology and Key Laboratory of Oral Biomedicine Ministry of Education, School and Hospital of Stomatology, Wuhan University, Wuhan, China; ^2^Shandong Provincial Key Laboratory of Oral Biomedicine, Department of Endodontics, School of Stomatology, Shandong University, Jinan, China

**Keywords:** DKK1, DLX3, human dental pulp cells, proliferation, Wnt/β-catenin signaling

## Abstract

Homeodomain gene *Distal-less-3* (*Dlx3*) plays an important role during tooth development. Our previous studies indicate that DLX3 inhibits proliferation of human dental pulp cells (hDPCs). However, the mechanism of DLX3 regulating proliferation of hDPCs and maintaining the quiescence of the cells remain unknown. Given the importance of canonical Wnt signaling in the proliferation of dental pulp cell and tooth development, we hypothesized that DLX3 inhibited proliferation of hDPCs through inactivation of canonical Wnt signaling. With overexpression or knock-down of DLX3 in primary hDPCs, we found DLX3 down regulated canonical Wnt signaling and its downstream target genes. And when the DLX3 overexpressed-cells were treated with lithium chloride, the proliferation inhibition by DLX3 was reversed. We also found that DLX3 enhanced the expression of DKK1 and the reduced proliferation of hDPCs by DLX3 was reversed with knock-down of DKK1. Furthermore, luciferase reporter assay and chromatin immunoprecipitation assay showed DLX3 was able to bind to *Dkk1* promoter region from nucleotides (nt) -1656 to -1245, and stimulated *Dkk1* promoter activity. Mutagenesis studies further revealed two DLX3 responsive elements in *Dkk1* promoter. Taken together, our data indicate that DLX3 inhibits proliferation of hDPCs via inactivation of Wnt/β-catenin signaling pathway by directly binding to *Dkk1* promoter and increasing its expression.

## Introduction

Dental pulp is mainly derived from neural crest cells and contains a mixed population of odontoblasts, fibroblasts, and undifferentiated stem/progenitor cells (Sinanan et al., 2004; Li et al., 2012). The main functions of dental pulp are dentinogenesis and maintaining the vitality of the dentin biologically and physiologically (Tatullo et al., 2015). Dentinogenesis is a progress involving the differentiation of preexisting undifferentiated stem/progenitor cells into odontoblasts and the formation of dentin. With mild or moderate dentin trauma, the secretion of dentin matrix by odontoblasts is increased and results in the formation of reactionary dentin that protects the pulp against damage. While with deep dentin lesions or injuries, original odontoblasts initially undergo cell death, and quiescent pulp stem/progenitor cells re-enter the cell cycle, proliferate and subsequently migrate to the pulp–dentin border behind injury region. Then these cells differentiate into a new generation of odontoblast-like cells, which secrete reparative dentin around the injury site and repair the dentin tissue (Harichane et al., 2011; Yan et al., 2011; Potdar and Jethmalani, 2015; Lv et al., 2016). However, under normal physiological conditions, as the only vascularized dental tissue that is encapsulated in rigid mineralized dentin structures, the dental pulp cells (DPCs) usually exhibit inhibited proliferation capacity (at quiescent state) (Yan et al., 2011), which makes homeostasis of the dental pulp (Gong et al., 2012). Many investigations were focused on the increased proliferation capacity of DPCs in response to decays or injuries, but the mechanism to maintain the very important inhibited proliferation ability of DPCs under normal condition is dismissed.

Distal-less3 (DLX3), as a transcription factor, is essential during embryo development (Morasso et al., 1999) and plays crucial roles in placental formation, epidermis development, and ectodermal appendages development (Morasso et al., 1995; Isaac et al., 2014). During early development, *Dlx3* is expressed in neural crest cells in branchial arches by E9.5 (Robinson and Mahon, 1994), and is firstly detected in dental epithelium and later extends to all dental area including epithelium and mesenchyme during the tooth morphogenesis (Zhao et al., 2000). In human, mutation of DLX3 is responsible for tricho-dento-osseous (TDO) syndrome, which is a rare autosomal dominant disorder and characterized by defects in hair, teeth, and other ectodermal appendages (Zhao et al., 2000; Hwang et al., 2008). In mice, deletion of *Dlx3* in neural crest severely impairs the odontoblast differentiation, dentin production, and transcription of *Dspp* (Duverger et al., 2012). Our previous study showed that DLX3 inhibited the proliferation of human DPCs (hDPCs) (Li et al., 2012). However, the molecular mechanism of DLX3 inhibiting hDPCs proliferation has not been elucidated.

Wnt signaling plays important role in regulating cell proliferation, differentiation, and polarity (Logan and Nusse, 2004; Rattanawarawipa et al., 2016). The canonical Wnt pathway mediates signaling through regulating intracellular level and subcellular localization of β-catenin. Without Wnt ligand, the enzyme glycogen synthase kinase 3β (GSK3β) will phosphorylate the cytoplasmic β-catenin, then the phosphorylated β-catenin will be further ubiquitylated and degraded (Rao and Kuhl, 2010). When Wnt ligand binds to its receptors, β-catenin will not be phosphorylated and the stabilized high level cytoplasmic β-catenin will be translocated into the nucleus. Then, a transcriptional complex will be formed by β-catenin and T-cell factor (Tcf)/lymphoid enhancer-binding factor (Lef) in the nucleus (MacDonald et al., 2009; Kim J.H. et al., 2013), which then activates expression of Wnt downstream genes such as CyclinD1, C-myc, and so on (Bejsovec, 2005; Liang et al., 2017). Inhibitory protein such as Dickkopf related protein 1 (DKK1) can inhibit Wnt signaling by preventing the binding of Wnt ligand with its receptor (Cruciat and Niehrs, 2013; Zhou et al., 2016).

Wnt/β-catenin signaling pathway plays important roles in different developmental stages of ectodermal appendages including teeth (Liu et al., 2008; Ahn et al., 2010). During tooth development, the expression of Lef1 and Fgf3 and the formation of primary enamel knot depend on the mesenchymal β-catenin (Chen et al., 2009; Du et al., 2012). Moreover, the Wnt/β-catenin signaling pathway has important functions on mineral matrix deposition (Aurrekoetxea et al., 2016) and odontogenic differentiation (Kim T.H. et al., 2013; Viale-Bouroncle et al., 2015). In mice, tooth development is arrested at bud stage due to the depletion of dental mesenchymal β-catenin (Sasaki et al., 2005). In human, mutation of *Wnt10a*, a canonical Wnt family member, has been shown to cause odonto-onycho-dermal dysplasia, an autosomal recessive ectodermal dysplasia characterized by defective tooth development (Adaimy et al., 2007). Previous study showed that WNT10A promoted the proliferation of hDPCs (Zhang et al., 2014). Thus, we proposed that DLX3 might inhibit proliferation and maintain the quiescent state of hDPCs by inhibiting Wnt signaling pathway.

In our study, we investigated the molecular mechanism of DLX3 in regulating hDPCs proliferation and found that DLX3 inhibited the proliferation of hDPCs through inactivation of Wnt signaling pathway via increasing DKK1 expression.

## Materials and Methods

### Isolation and Culture of hDPCs

Dental pulp tissues were obtained from extracted healthy human premolars for orthodontic treatment (12–14 years old) with informed consents. All the procedures were approved by the Ethics Committee of the School of Stomatology, Wuhan University. After extraction, the tooth was washed three times with phosphate-buffered saline solution (PBS, Invitrogen, Carlsbad, CA, United States). The pulp tissue was gently separated from the teeth and cut into small pieces (approximately 1 mm^3^ in size). The tissue pieces were seeded into a T25 cell culture bottle in Dulbecco’s Modified Eagle Medium (DMEM, Gibco-BRL, Grand Island, NY, United States) supplemented with 10% fetal bovine serum (FBS, Gibco-BRL) and antibiotics. Upon reaching confluence, cells were passaged at a threefold dilution. The cells between passage 2 and 6 were used in this study. For lithium chloride (LiCl) treatment, hDPCs were pre-treated with 20 mM LiCl (Sigma, St. Louis, MO, United States) for 24 h before collecting.

### Lentiviral Infection

To continuously overexpress DLX3, hDPCS were infected with lentivirus using pLL3.7-Dlx3 plasmid and packaging plasmids as described previously (Li et al., 2012). Cells successfully infected with *Dlx3* lentiviral particles were assigned as hDPC/*Dlx3*. Control cells infected with empty vector and wild-type hDPCs were assigned as hDPC/*pLL3.7* and hDPC/*wt*, respectively.

### Western Blot

Cells were harvested and lysed for total protein in RIPA lysis buffer (Thermo Scientific, EU, Lithuania) with protease inhibitors. Proteins were separated by sodium dodecyl sulfate polyacrylamide gel (SDS-PAGE) and subsequently transferred to polyvinylidene fluoride (PVDF) membrane. Membranes were then incubated with anti-DLX3, anti-CyclinD1, anti-C-myc, anti-Tcf-7, anti-DKK1, anti-β-actin, anti-GAPDH (all from Abcam, Cambridge, MA, United States), and anti-active-β-catenin antibodies (Millipore, Burlington, MA, United States) overnight at 4°C. Horseradish peroxidase (HRP)-conjugated secondary antibodies (1:10,000) and chemiluminescence substrate were used to detect the immunoreactive bands with autoradiography film. Protein levels were quantitatively analyzed with Image J (National Institutes of Health, Bethesda, MD, United States) with β-actin as the loading control.

### Quantitative Real-Time PCR (qPCR)

Total RNAs were isolated with EZNA Total RNA Kit I (Omega, Norcross, GA, United States) and were reverse transcribed by using Revert Aid First Strand cDNA Synthesis Kit with gDNA Eraser (Thermo Scientific). qPCR was conducted with the ABI Prism 7500 Real-Time PCR System (Life Technologies, Foster City, CA, United States) with SYBR green master mix (Roche Diagnostics, Indianapolis, IN, United States). Specificity of qPCR in each sample was confirmed by melting curve analyses. The relative mRNA expression was normalized to the control glyceraldehyde-3-phosphate dehydrogenase (*Gapdh*). The primers designed for *Dlx3*, *Dkk1*, *CyclinD1*, *C-myc*, and *Gapdh* are listed in Table 1.

**Table 1:** Primer sequences used for qPCR amplification.

Gene	Sequence (5′-3′)	Size (bp)
*Dlx3*	Forward: CCTATGGCCAGACGGTGAAC	222
	Reverse: GCACTCCTCGTCCTCTG	
*Dkk1*	Forward: CTCGGTTCTCAATTCCAACG	120
	Reverse: TTTCTGTTGCCACTGCTGGGAC	
*C-myc*	Forward: CCACACATCAG ACAACTACGCT	100
	Reverse: GCATTTTCGGTTGTTGCTGATC	
*CyclinD1*	Forward: AGGTCTGCGAGGAACAGAAGTG	137
	Reverse: TGCAGGCGGCTCTTTTTC	
*Gapdh*	Forward: TCATGGGTGTGAACCATGAGAA	146
	Reverse: GGCATGGACTGTGGTCATGAG	

### Immunofluorescence Staining

Cells seeded on coverslips were cultured for 24 h after attachment. After washing with cold PBS, cells were fixed with 4% paraformaldehyde for 15 min at room temperature, permeabilized with 0.25% Triton X, and blocked in 3% BSA at 37°C for 1 h. Cells were finally incubated with anti-active-β-catenin or anti-DKK1 primary antibody at 4°C overnight. Then cells were incubated with Alexa Fluor 488- or 594-conjugated secondary antibody (Abcam), followed by counterstaining with 4′,6-diamidino-2-phenylindole (DAPI).

### EdU Staining

The proliferation ability of cells was assessed by EdU Staining with the Cell-Light EdU DNA cell proliferation kit (RiboBio, Guangzhou, China). Briefly, hDPCs cultured in 24-well plate were incubated with 30 μM 5-ethynyl-2′-deoxyuridine (EdU) for 3 h. Nuclei were stained with DAPI. The EdU-positive cells and total cells were calculated and the ratio of EdU-positive cells to total cells was statistically analyzed between groups.

### RNA Interference

To knock-down the endogenous DLX3 or DKK1 expression, small interference RNA (siRNA) targeted human *Dlx3* (*Dlx3* siRNA, GenePharma, Suzhou, China) or human *Dkk1* (*Dkk1* siRNA, Sigma) or negative control siRNA (Neg. siRNA, Sigma) were transfected into cells at a final concentration of 40 nM with Lipofectamine 2000 (Invitrogen). Forty-eight hours after transfection, the cells were subjected to Western blot or qPCR or EdU staining. Cells transfected with *Dlx3* siRNA were assigned as hDPC/*Dlx3 si*.

### Construction of Reporter Plasmids and Mutagenesis

The human *Dkk1* promoter region from -1656 to +398 relative to the position of the transcription start site (+1) was amplified by PCR using human genomic DNA as template with the following primer pairs (*p1656*): forward: 5′-cgcctcgagCTACTCG AGCTATAGTAGGTCAGCATGTGGAGTC-3′; reverse: 5′-cgcaa gcttACGAAGCTT AGGTCAGAGCATCCTCTGAGT-3′. Other fragments with different lengths in the 5′-flanking region were amplified by PCR using *p1656* as template with the same reverse primer and the following forward primers: *p1245*, 5′-cgcctcgagCTACTCGAG GGCTCTAGGCTTCCAATAAGT-3′; *p845*, 5′-cgcctcgagCTACTCGAGCTGCCTA ATCAAGTTCATCTACCG-3′. All the PCR forward primers and reverse primers contain a 5′-cgcctcgag-*XhoI* overhang and a 5′-cgcaagctt-*HindIII* overhang, respectively. All the PCR products were gel-purified and finally subcloned into the pGL3-Basic vector (Promega, Madison, WI, United States) after digesting with *HindIII* and *XhoI* (New England Biolabs, Ipswich, MA, United States). To construct the mutant reporter plasmids (*Dlx3I Del*, *Dlx3II Del*, *Dlx3III Del*, and *Dlx3IV Del*), QuikChange II Site Directed Mutagenesis Kit (Agilent Technologies, Santa Clara, CA, United States) was used with *p1656* as template and following oligonucleotide primers: *Dlx3I Del*, forward 5′-TAAATAT ATCTTATCTTTTTCAATAGTGATTATTCAATCAC-3′, reverse 5′-GTGATTGAA TAATCACTAT TGAAAAAAGTAAGATATATTTA-3′, *Dlx3II Del*, forward 5′-AT ATCAACCTGTTTTTAAAAGTG TATGCTT-3′, reverse 5′-CAATAAATATAAAA GCATACACTTTTAAAAACAGGTTGATAT-3′, *Dlx3III Del*, forward 5′-ATGCT TTTATATTTATTGCTATTATTGTTACTACATCTTTTA-3′, reverse 5′-TAAAAG ATGTAGTAACAATAATAGCAATAAATATAAAAGCAT-3′, and *Dlx3IV Del*, forward 5′-CTACATCTTTTATTATTACTGGAATAAAGAGAACTTTATTAT-3′, reverse 5′-ATAATAAAGTTCTCTTTATTCCAGTAATAATAAAAGATGTAG-3′. All mutant constructs were verified by DNA sequencing.

### Dual Luciferase Reporter Assay

293T cells grown in 48-well plates were transfected with pGL3-Basic vector or the reporter plasmids, or mutant reporter plasmids and pRL-TK *Renilla* luciferase expression vector (Promega) as an internal control as well as human DLX3 overexpressing plasmid (pcDNA3.1-Dlx3). After 48 h, the cells were collected and lysed. Luciferase activities were measured according to the Dual Luciferase reporter assay system (Promega). Relative luciferase activities were determined by normalizing each *Firefly* luciferase activities to *Renilla* luciferase activities. All experiments were conducted at least three times.

### Chromatin Immunoprecipitation (ChIP)

293T cells were co-transfected with *p1656* with or without pcDNA3.1-Dlx3 for 48 h. Chromatin immunoprecipitation (ChIP) assay was conducted with the ChIP assay kit (Upstate Technology, Richfield Springs, NY, United States) as described previously (Yang et al., 2017). Anti-DLX3 antibody or negative control IgG was used for immunoprecipitation. The purified immunoprecipitated DNA was analyzed by PCR to amplify promoter regions of the *Dkk1* gene using the following primers: forward ^-1656^5′-CTATACAGCTTAGAGGAAGACAAAATA-3^′-1230^, reverse ^-1271^5′-CAAAT ATTTATGAGACACAGCTCTCAT-3^′-1245^. The input DNA from samples before immunoprecipitation was used for the positive control.

### Statistical Analysis

Each experiment was conducted at least three times. The data were presented as means ± standard deviation. Statistical analyses were conducted by one-way analysis of variance by using the GraphPad Prism, PC version 7 (GraphPad software). *P* < 0.05 was considered statistically significant.

## Results

### DLX3 Inactivates Canonical Wnt/β-Catenin Pathway in hDPCs

**FIGURE 1:**
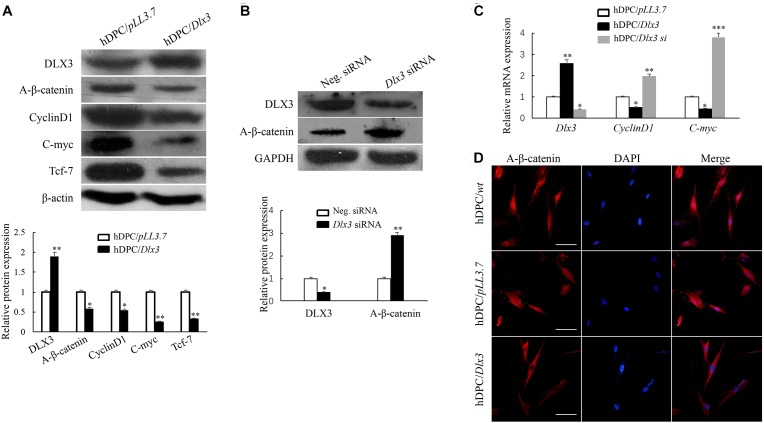
DLX3 inactivates canonical Wnt/β-catenin pathway in hDPCs. **(A)** Western blot showed the expressions of DLX3, active β-catenin (A-β-catenin), CyclinD1, C-myc, Tcf-7 and β-actin in hDPC/*pLL3.7* and hDPC/*Dlx3* cells. Lower part shows the quantitative analysis. **(B)** Western blot showed the expression of DLX3 and active β-catenin in hDPCs transfected with negative control siRNA or *Dlx3* siRNA. Lower part shows the quantitative analysis. **(C)** qPCR showed the relative mRNA expressions of *Dlx3*, *CyclinD1* and *C-myc* in hDPC/*pLL3.7*, hDPC/*Dlx3* and hDPC*/Dlx3 si* cells. ^∗^*P* < 0.05, ^∗∗^*P* < 0.01, ^∗∗∗^*P* < 0.001. **(D)** Immunofluorescence staining showed the subcellular localization of active β-catenin in hDPC/*wt*, hDPC/*pLL3.7* and hDPC/*Dlx3* cells. Scale bars = 50 μm.

To evaluate whether DLX3 inactivates Wnt/β-catenin signaling pathway in hDPCs, active β-catenin, an indicator of canonical Wnt signaling activity, was detected by Western blot. As shown in Figure 1A, the expression of active β-catenin was decreased in hDPC/*Dlx3* cells. CyclinD1, C-myc and Tcf-7 were known as target genes of canonical Wnt/β-catenin signaling pathway (Arya et al., 2015; Kaur et al., 2015). As expected, the expressions of CyclinD1, C-myc, and Tcf-7 were also decreased in hDPC/*Dlx3* cells (Figure 1A). Then, the endogenous DLX3 in hDPCs were knocked down with *Dlx3* siRNA, and the expression of active β-catenin was increased (Figure 1B). qPCR showed the similar results to that of Western blot (Figure 1C). To further confirm the inhibition of canonical Wnt/β-catenin by DLX3, the subcellular localization of active β-catenin in hDPCs were detected by immunofluorescence staining. In hDPC/*wt* and hDPC/*pLL3.7* cells, active β-catenin was highly expressed in both cytoplasm and nucleus (Figure 1D). However, in hDPC/*Dlx3* cells active β-catenin was only expressed in cytoplasm but not in nuclei (Figure 1D). All these results indicated that DLX3 inactivated the canonical Wnt/β-catenin signaling in hDPCs.

### DLX3 Inhibits Proliferation of hDPCs Through Inactivation of Wnt Signaling

**FIGURE 2:**
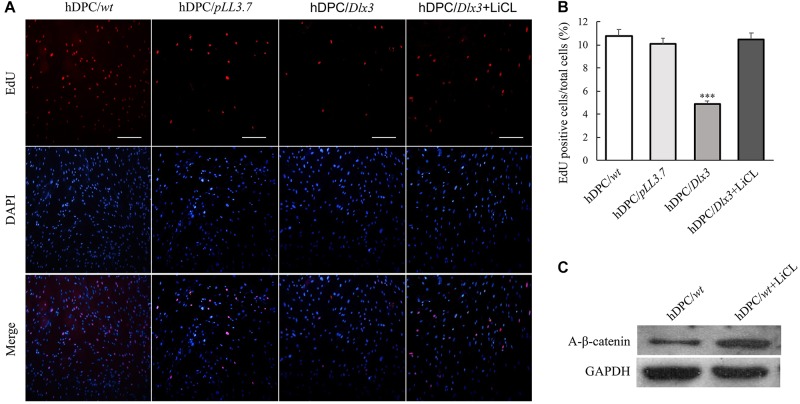
DLX3 inhibits proliferation of hDPCs through inactivation of Wnt signaling. **(A)** The cell proliferation ability was assessed by EdU staining in hDPC/*wt*, hDPC/*pLL3.7*, and hDPC/*Dlx3* cells with or without pretreatment of 20 mM LiCl. Scale bars = 200 μm. **(B)** Quantitative analysis (the ratio of the EdU-positive cell to the total cell number) of the EdU staining in **A**. ^∗∗∗^*P* < 0.001. **(C)** Western blot showed the expression of active β-catenin in cells with or without LiCl treatment.

LiCl is able to activate canonical Wnt/β-catenin signaling by inhibiting GSK-3β (Hedgepeth et al., 1997). To further test whether DLX3 inhibits proliferation of hDPCs via inactivation of Wnt signaling, cells were treated with 20 mM LiCl. Then the cell proliferation was measured with EdU labeling assay. The cell proliferative rate in hDPC/*Dlx3* cells was dramatically suppressed compared with that in hDPC/*wt* and hDPC/*pLL3.7* cells, but was significantly reversed when cells were treated with LiCl (Figures 2A,B), which suggested that DLX3 inhibited the proliferation of hDPCs through inactivation of Wnt signaling pathway. In addition, Western blot confirmed the activation of canonical Wnt/β-catenin signaling with LiCl treatment by detecting the expression of active β-catenin (Figure 2C).

### DLX3 Inhibits Proliferation of hDPCs via Increasing DKK1 Expression

**FIGURE 3:**
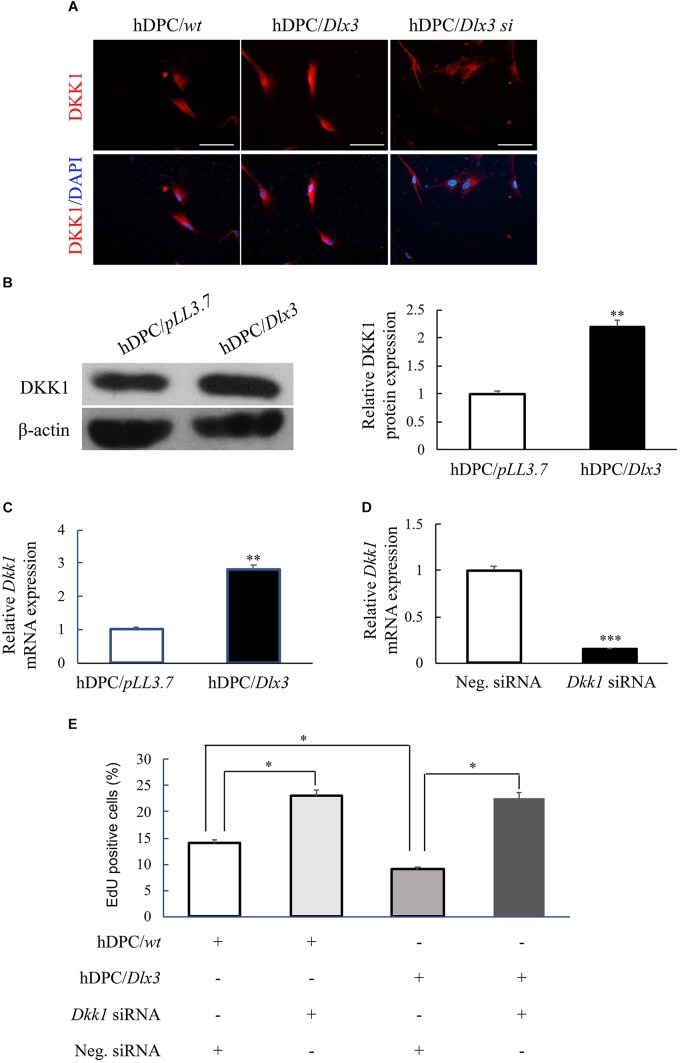
DLX3 inhibits proliferation of hDPCs via increasing DKK1 expression. **(A)** The expressions of DKK1 in hDPC/*wt*, hDPC/*Dlx3*, and hDPC/*Dlx3 si* cells were detected by immunofluorescence staining. Scale bars = 50 μm. **(B,C)** Western blot and qPCR showed the protein and mRNA expression of DKK1 in hDPC/*pLL3.7* and hDPC/*Dlx3* cells. **(D)** qPCR showed the relative *Dkk1* mRNA expression in hDPCs transfected with negative control siRNA or *Dkk1* siRNA. **(E)** Cell proliferation ability was assessed by EdU staining in hDPC/*wt* or hDPC/*pLL3.7* cells with transfection of negative control siRNA or *Dkk1* siRNA. ^∗^*P* < 0.05, ^∗∗^*P* < 0.01, ^∗∗∗^*P* < 0.001.

Immunofluorescence staining showed that the expression of DKK1, an antagonist of Wnt signaling pathway, was increased in hDPC/*Dlx3* cells, and decreased in hDPC/*Dlx3 si* cells, compared with that in hDPC/*wt* cells (Figure 3A). Western blot and qPCR results also showed that overexpression of DLX3 enhanced DKK1 expression in hDPCs (Figures 3B,C). To further investigate whether DLX3 inhibits proliferation of hDPCs via increasing DKK1 expression, *Dkk1* siRNA was transfected into hDPC/*Dlx3* cells or hDPC/*wt* cells. The knock-down efficiency of *Dkk1* siRNA was confirmed by qPCR, which showed the expression of endogenous *Dkk1* was reduced more than 70% when cells transfected with *Dkk1* siRNA compared with cells transfected with control siRNA (Figure 3D). EdU staining showed that the reduced proliferation of hDPCs by DLX3 overexpression was reversed with knock-down of DKK1 (Figure 3E). All these results indicated that DLX3 inhibited the proliferation of hDPCs through downregulation of Wnt/β-catenin signaling pathway via increasing DKK1 expression.

### DLX3 Increases DKK1 Expression by Directly Stimulating *Dkk1* Promoter Activity

**FIGURE 4:**
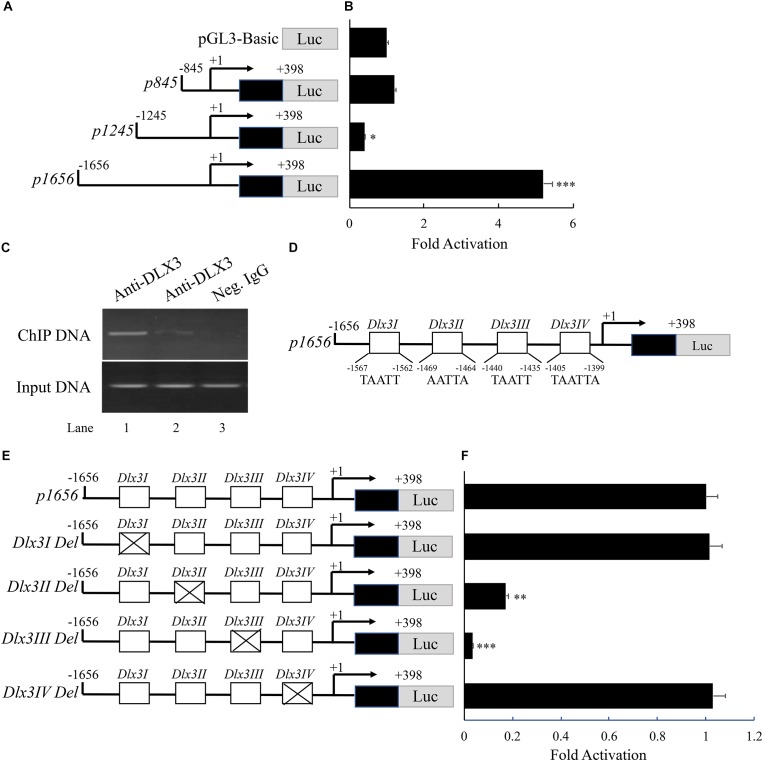
DLX3 increases DKK1 expression by directly stimulating *Dkk1* promoter activity. **(A)** Schematic representation of reporter constructs with different lengths of *Dkk1* promoter. **(B)** 293T cells were co-transfected with pGL3-Basic or *p845* or *p1245* or *p1656* as well as pcDNA3.1-Dlx3 plasmids. Luciferase activity was determined. ^∗^*P* < 0.05, ^∗∗∗^*P* < 0.001, compared with pGL3-Basic group. **(C)** ChIP assay demonstrated the binding of DLX3 with *Dkk1* promoter. Neg. IgG, Negative control IgG. **(D)** Illustration of core sequence of four DLX3 responsive elements: *Dlx3I*, *Dlx3II*, *Dlx3III*, and *Dlx3IV*. **(E)** Schematic representation of wild type and mutant *p1656* reporter constructs. **(F)** 293T cells were co-transfected with wild type or mutant *p1656* reporter constructs as well as pcDNA3.1-Dlx3 plasmid. Luciferase activity was determined. ^∗∗^*P* < 0.01, ^∗∗∗^*P* < 0.001, compared with wide type *p1656* group.

To assess whether induction of DKK1 expression is through directly stimulating *Dkk1* promoter activity, luciferase reporters containing different lengths of *Dkk1* promoters, *p845*, *p1245*, and *p1656*, were constructed (Figure 4A) and transfected into 293T cells. Luciferase activity was measured with co-transfection of pcDNA3.1-Dlx3 vector (Figure 4B). Overexpression of DLX3 failed to increase transcription activates of *p845* and *p1245* compared with transfection of pGL3-Basic, but significantly increased the transcription activity of *p1656*, which suggested DLX3 was able to stimulate *Dkk1* promoter activity from nucleotides (nt) -1656 to -1245. To further confirm the direct binding of DLX3 with nt -1656 to -1245 of *Dkk1* promoter *in vivo*, ChIP assay was performed. PCR was conducted with the immunoprecipitated and purified DNA as template and the oligonucleotides corresponding to the 5-flanking region (from nt -1656 to -1245) of *Dkk1* promoter as primers. As expected, PCR band was detected (Figure 4C, Lane 2, upper band) and was more intense when the cells were co-transfected with pcDNA3.1-Dlx3 plasmid (Figure 4C, Lane 1, upper band). Positive control was set using input DNA as template (Figure 4C, lower bands), and negative control was set using negative control IgG (Figure 4C, Lane 3). These results indicated that DLX3 is able to directly bind to *Dkk1* promoter region from nt -1656 to -1245, and stimulates *Dkk1* promoter activity.

It is known DLX3 binds to a conserved sequence of TAATT (Feledy et al., 1999). Analysis of the promoter region from nt -1656 to -1245 of *Dkk1* gene revealed four potential DLX3 responsive elements (*Dlx3I*, *Dlx3II*, *Dlx3III*, and *Dlx3IV*; Figure 4D). To further confirm whether DLX3-induced *Dkk1* promoter activity is mediated by these potential responsive elements, four *p1656* mutant constructs with deletion of DLX3 responsive elements were generated (Figure 4E). *p1656* or *p1656* mutant reporter constructs with pcDNA3.1-Dlx3 plasmid were co-transfected into 293T cells. Luciferase reporter assay showed that deletion of either *Dlx3I* or *Dlx3IV* did not change the response activity of *p1656* to DLX3, but deletion of either *Dlx3II* or *Dlx3III* did significantly suppress the response activity of *p1656* to DLX3 (Figure 4F), which indicated that DLX3-induced *Dkk1* promoter activity is mediated by *Dlx3II* and *Dlx3III* elements.

## Discussion

Our present investigation revealed the underlying mechanism of DLX3 controlling the proliferation and maintaining quiescence of hDPCs. DLX3 is able to enhance DKK1 expression in hDPCs, then suppresses Wnt/β-catenin signaling and inhibits the proliferation of hDPCs.

DKK1 is a typical antagonist of Wnt/β-catenin signaling by competing for the Wnt receptor lipoprotein receptor related protein (LRP)5/6 (Kawano and Kypta, 2003). During mouse molar morphogenesis, *Dkk1* is primarily expressed in dental mesenchyme, including preodontoblasts (Fjeld et al., 2005). Postnatally, *Dkk1* is prominently expressed in the preodonto- and odonto-blasts (Fjeld et al., 2005). In *Dkk1* transgenic mice, with overexpression of *Dkk1* in dental pulp and odontoblasts, the molar shows an increase of immature odontoblasts, few mature odontoblasts and dramatic change in Osx and nestin expression (Han et al., 2011). The present investigation found the enhanced DKK1 in hDPC/*Dlx3* cells suppressed Wnt/β-catenin signaling and inhibited the proliferation of hDPCs. These results are consistent with previous studies, which showed that the DKK1 inhibited the proliferation of several types of cells, including human retinal pigment epithelial cells, periosteal cells, osteosarcoma cells, via downregulation of Wnt/β-catenin signaling pathway (Kim H.K. et al., 2013; Liu et al., 2014; Zhou et al., 2016).

We identified *Dkk1* as a target gene of DLX3, which is supported by the increased expression level of DKK1 in hDPC/*Dlx3* cells. To further elucidate the mechanism of upregulation of the DKK1 expression by DLX3, a series of tests focused on *Dkk1* promoter was performed. Luciferase reporter assay showed the transcription activity of *p1656* was significantly enhanced compared with that of *p1245*, *p845*, and pGL3-Basic vector with transfection of DLX3 expression vector, which indicated DLX3 can activate *Dkk1* promoter from nt -1656 to -1245. Then we found either endogenous or overexpressed DLX3 can bind with this promoter region using ChIP assay, and four potential DLX3 response elements were found in this region. Furthermore, with mutation of each binding elements, we found only two DLX3 response elements (*Dlx3 II* and *Dlx3 III*) are functional and essential for transcriptional activation of *Dkk1* promoter. These studies clearly demonstrated the regulation mechanism of DKK1 expression by DLX3.

Previous studies showed many factors, such as growth and differentiation factor-5 (GDF5), insulin-like growth factor, Wnt10A, and Wnt3A, can enhance the proliferation of hDPCs (Nemoto et al., 2009; Chang et al., 2013; Zhang et al., 2014; Lv et al., 2016). Although the increased proliferation capacity of hDPCs has important implications during regeneration, the hDPCs are usually in a quiescent state in dental pulp under normal conditions (Yan et al., 2011). Thus we investigated the mechanism of how hDPCs maintaining its quiescent state. Wnt signaling can be subdivided into canonical and non-canonical pathways. Non-canonical Wnt signaling antagonizes canonical Wnt signaling (Mikels and Nusse, 2006). Non-canonical Wnt signaling maintains quiescent state of hematopoietic stem cells (HSC) (Sugimura et al., 2012). Along with the activation of HSCs, non-canonical Wnt signaling was attenuated and canonical Wnt signaling was enhanced (Sugimura et al., 2012). During hair follicle growth, canonical Wnt signaling is generally believed to be inactive in the quiescent telogen bulge (Hsu and Fuchs, 2012), and when bulge cells are activated to undergo the transition from telogen to anagen, canonical Wnt signaling becomes strongly elevated (Lien et al., 2014). In β-catenin conditional knockout mice, hair follicle stem cells (HFSCs) remained quiescent and showed no signs of anagen re-entry (Lien et al., 2014). In the cell cycle of myoblasts, canonical Wnt inhibitors Dkk3 and Rgs2 were more strongly induced during G0 entry, and rapid suppression upon cell cycle re-entry (Subramaniam et al., 2014), which indicates cells in a canonical Wnt-rich environment does not hold for quiescence. Based on these researches, we inferred the inhibited canonical Wnt signaling might play important role in quiescence of hDPCs. In our present study, we found DLX3 inhibited the proliferation of hDPCs and maintained quiescence of the cells indeed through inactivation of canonical Wnt/β-catenin signaling pathway, which is confirmed by the evidence that LiCl treatment reversed the inhibited proliferation ability of hDPC/*Dlx3* cells.

Above all, in the present investigation we elucidate the mechanism of DLX3 in inhibiting proliferation and maintaining the quiescence of hDPCs, which will help to understand the homeostasis of dental pulp in normal physiological conditions.

## Author Contributions

YZ and XL performed most of the experiments, collected and analyzed the data, and prepared the figures. XG performed some of the experiments. MF provided advice and revised the manuscript. GYu and GYa designed the experiments, interpreted the experimental results, and drafted the manuscript. All authors approved the final version of the manuscript.

## Conflict of Interest Statement

The authors declare that the research was conducted in the absence of any commercial or financial relationships that could be construed as a potential conflict of interest.
